# The relationship between preoperative blood pressure during anesthetic examinations and pre-intubation blood pressure

**DOI:** 10.1186/s12871-024-02477-x

**Published:** 2024-03-02

**Authors:** Ikuya Koibuchi, Yuji Kadoi, Chizu Asou, Shigeru Saito

**Affiliations:** 1 Department of Anesthesiology, Gunma University Graduate School of Medicine, 3-39-22 Showa-Machi, Maebashi, Gunma 371-8511 Japan; 2 Division of Operation Room, Gunma University Hospital, 3-39-15 Showa-Machi, Maebashi, Gunma 371-8511 Japan

**Keywords:** Preoperative examination, Blood pressure, Repeated measure, Pre-intubation/induction, White-coat hypertension

## Abstract

**Background:**

There have been few reports showing the relationship between blood pressure (BP) measured at clinics preoperatively and BP measured before anesthetic intubation/induction. The purpose of this study was to examine the relationship between BP measured at different times and settings preoperatively and BP measured before intubation/induction.

**Methods:**

A total of 182 patients who underwent general anesthesia between March 2021 and April 2022 in a university hospital were examined. In addition to self-reported BP asked on an anesthetic examination sheet completed by each patient, BPs were measured three times, before, during, and after preoperative examination by the anesthesiologist. The derived parameter was compared with BP measured before intubation at the time of general anesthesia induction.

**Results:**

The systolic BP in the intra-examination period had the most significant correlation with pre-intubation systolic BP (*r* = 0.5230, *p* < 0.0001, 95% CI = 0.4050 to 0.6238). On Bland–Altman analysis, the intra-examination systolic BP seemed to be similar and showed better agreement with pre-intubation systolic BP than other measured BPs, with a mean bias of 2.2 mmHg and the narrowest 95% limits of agreement (-33.7 to + 38.1 mmHg).

**Conclusions:**

The preoperative systolic BP value measured during the examination by the anesthesiologist was found to be closely related to pre-intubation systolic BP measured in the operating room. Higher BP during the preoperative examination may be a result of anxiety-induced stress or white-coat hypertension. Measuring BP during the anesthesiologist’s examination may be useful for predicting hypertension in the pre-intubation period.

## Introduction

Stabilizing blood pressure (BP) in the perioperative period is one of the most important issues for anesthesiologists. When anesthesiologists plan their BP management strategy, preoperative BP or “baseline BP” is essential information for managing the BP level in the perioperative period [1].

In 1980, Bedford et al. [2] reported that hypertension at the time of hospital admission was a predictor of more severe reactive hypertension following rapid-sequence tracheal intubation. In 2017, Venkatesan et al. [3] reported that there was a significant association between preoperative diastolic BP values and increased postoperative 30-day mortality in elderly persons. These reports suggest that obtaining a precise BP for each patient in the preoperative period is crucial to planning and maintaining anesthesia. However, due to the shorter length of hospital stay and limited time for preoperative examinations by anesthesiologists, it is relatively difficult to obtain precise BP measurements for each patient preoperatively. It is common knowledge that many patients undergoing surgery are admitted to hospital on the day before or on the day of the surgery, and anesthesiologists have little time to examine the patient after admission due to the patients’ other preparations.

BP measured before anesthetic intubation/induction, or pre-intubation BP, is the easiest to refer to as baseline BP, but it is widely known that it is relatively higher than daily BP measured at home because of anxiety-induced stress before the surgery [4–7]. Drummond et al. [8] showed the effect of “white-coat hypertension” on the day of surgery compared to baseline BP measured at home. If anxiety-induced stress or white-coat hypertension affects the pre-intubation BP as reported, we thought that preoperative examinations by anesthesiologists could have a similar effect on the preoperative BP measured at clinics.

Until now, there have been few reports showing the relationship between the preoperative BP measured at clinics and the pre-intubation BP for general anesthesia [5–7]. Van Klei et al. [5] examined the relationship between preoperative BP measured before the day of surgery and the pre-intubation BP, and they showed that the average pre-intubation BP was higher than the preoperative BP. However, previous reports compared only one or two points of BP measurement in the preoperative period, and it did not examine the effect of anxiety-induced stress or white-coat hypertension on preoperative BP.

Therefore, the purpose of this study was to examine the relationship between pre-intubation BP and BP measured at different times and settings in the preoperative period, especially before, during, and after anesthesiologists’ examinations, and whether anxiety-induced stress or white-coat hypertension could influence preoperative BP values.

## Methods

### Study design

This was a retrospective observational study that evaluated cardiovascular parameters during preoperative examinations and anesthesia induction. Patients who underwent general anesthesia between 1 March 2021 and 30 April 2022 in a university hospital were included.

The authors obtained approval from the Human Ethics Committee of their institute (HS2022-071) and registered the study protocol at UMIN-CTR (#000050350).

### Data acquisition

Prior to the preoperative examination by the anesthesiologists, patients reported their “daily measured BP” or “remembered most recently measured BP” on an anesthetic examination sheet as their “self-reported BP”. BP and heart rate (HR) were measured three times, before, during, and after the preoperative examination (pre-examination BP/HR, intra-examination BP/HR, and post-examination BP/HR). At the hospital, BP and HR were measured by a standard upper arm BP monitor (HBP-9030, Omron, Tokyo, Japan) before and after preoperative examination and by a wrist BP monitor (EW-BW10-W, Panasonic, Tokyo, Japan) during the preoperative examination. Information about the devices used in patients’ daily blood pressure measurements was not collected in this study.

The subjects sat on a bench seat and placed their arm on a pillow to elevate their wrist to the level of the mid sternum while the measurements were taken. This position was suggested by the manufacturer for proper measurement, and the sitting position effectively stabilized the bodies of the participants during measurement. Additional medical information for the patients: sex, age, body weight, past medical history, and pre-operative test results (blood test, chest X-ray, electrocardiogram, etc.) were gathered as part of the normal assessment before anesthesia.

During the anesthesia procedure, BP and HR were measured by a standard upper arm BP monitor and electrocardiogram monitor with the patient in the supine position. The methods of anesthesia induction and maintenance were not specified. The measured BP and HR before intubation/induction for general anesthesia (pre-intubation BP/HR) were collected postoperatively from the anesthesia record. In addition, information about the anesthesia method and surgery type were gathered postoperatively. Correlations between the preoperatively collected systolic BPs (self-reported BP, pre-examination BP, intra-examination BP, and post-examination BP) and pre-intubation systolic BP were examined. Agreement between the two blood pressure measurements was evaluated by Bland–Altman analysis.

As a subgroup analysis, the patients were divided into two groups, with or without regular antihypertensive medication, and the same comparison and analysis for measured systolic BPs were performed to see if antihypertensive medication affects the results.

Whether patients’ self-reported BPs are more reliable if they have daily measurements was also examined. The patients were divided into two groups: one with no BP measuring devices at home and whose BPs were only measured at their annual health check occasionally, and the other with BP measuring devices at home and who measured BP regularly. Whether there was a significant difference between self-reported systolic BP and pre-examination BP was examined in these two groups, and whether there was a stronger correlation between self-reported systolic BP and pre-examination BP for each group was also evaluated.

### Statistics

All data are expressed as median ± interquartile range (IQR) values. Statistical comparisons of the measured blood pressures were assessed by Friedman’s test followed by Dunnett’s multiple comparisons test. Correlations between each value were assessed with Spearman’s rank-correlation coefficient. A Bland–Altman analysis was performed to assess the agreement between two blood pressure measurements. Statistical software (GraphPad Prism version 9.3.1 for Windows, GraphPad Software, Boston, MA, USA, www.graphpad.com) was used in these tests and analyses. A *p* value < 0.05 was considered significant. The data are reported according to the STROBE statement. The sample size provides 80% power to detect a potentially clinically relevant blood pressure difference between the preoperative sBP and the pre-induction sBP of at least 15 mmHg, with a 5% probability of a type 1 error. As a reference, in a previous study which consists of 3360 patients [5], there was a statistically significant correlation between preoperative and preinduction mean BP values with a Pearson correlation coefficient of 0.43 (95% confidence interval: CI = 0.40 to 0.45).

## Results

Table 1 shows the participants’ demographic data. The study cohort included 182 adult patients, of whom 82 used antihypertensive medication regularly.

**Table 1:** Participants’ demographic characteristics

N = 182	Median (IQR)	Number of Patients (%)
Age (y)	69 (56.2–75.7)	
< 70		95 (53.8%)
≥ 70		87 (46.1%)
Sex	Male	98 (52.2%)
	Female	84 (47.8%)
Height (cm)	161 (154–167)	
Weight (kg)	58 (50–68)	
Body mass index (kg/m^2^)	22.7 (20.4–25.3)	
Diabetes mellitus (DM)	Non-DM	140 (76.9%)
	Diet only (without medication)	9 (4.9%)
	Oral treatment	21 (11.5%)
	Insulin user	12 (6.6%)
Hypertension (HT)	Non-HT	82 (45.1%)
	Borderline (without medication)	18 (9.9%)
	Oral treatment	82 (45.1%)
Hyperlipidemia (HL)	Non-HL	122 (67.0%)
	Borderline (without treatment)	9 (4.9%)
	Oral treatment	51 (28.0%)
Smoking within 1 month	No	159 (87.3%)
	Yes	23 (12.6%)
Chronic kidney disease (CKD)	Non-CKD	137 (75.3%)
	Mild-moderate	44 (24.1%)
	Severe (on hemodialysis)	1 (0.5%)
History of arteriosclerotic disease	Never	160 (87.9%)
	Yes	22 (12.1%)
ASA-PS	1	7 (3.8%)
	2	149 (81.9%)
	3	26 (14.3%)

Data are expressed as median (Interquartile range: IQR) values

*ASA* American Society Anesthesiology, *PS* Physical Status

Table 2 shows the BP changes at each time point. The median pre-intubation systolic BP was almost similar to the median intra-examination systolic BP. Most systolic BP measurements showed a significant difference between each time point (self-reported vs. pre-examination, *p* < 0.0001; self-reported vs. intra-examination, *p* < 0.0001; self-reported vs. post-examination, *p* = 0.0009; self-reported vs. pre-intubation, *p* < 0.0001; pre-examination vs. intra-examination *p* < 0.0001; pre-examination vs. post-examination, *p* = 0.0021; pre-examination vs. pre-intubation, *p* < 0.0001; intra-examination vs. post-examination, *p* < 0.0001; post-examination vs. pre-intubation, *p* < 0.0001), except for between systolic BP measured in the intra-examination period and the pre-intubation period (intra-examination vs. pre-intubation *p* > 0.9999).

**Table 2:** Blood pressure (BP) and heart rate (HR) values for each setting and time point

Measurement point		Median (IQR)
Self-reported BP on examination sheet	Systolic/Diastolic BP (mmHg)	130/70 (118.5–135/65–80)
	Mean BP (mmHg)	90 (83.3–96.6)
BP before anesthesiologists’ examination (Pre-)	Systolic/Diastolic BP (mmHg)	137/76 (124.2–149/67–83)
	Mean BP (mmHg)	96.6 (87.0–104.2)
	HR (bpm)	73 (66–81)
BP during anesthesiologists’ examination (Intra-)	Systolic/Diastolic BP (mmHg)	142.5/87 (131.2–159/79.2–97.7)
	Mean BP (mmHg)	105.5 (98.6–115.2)
	HR (bpm)	73 (67.2–81)
BP after anesthesiologists’ examination (Post-)	Systolic/Diastolic BP (mmHg)	130.5/74.5 (122–144/67–81)
	Mean BP (mmHg)	93.3 (86–102.3)
	HR (bpm)	71 (65–79.7)
BP at pre-anesthesia induction in operating room	Systolic/Diastolic BP (mmHg)	146/86 (134–163/77–93)
	Mean BP (mmHg)	106 (96.4–114.9)
	HR (bpm)	72.5 (64–83)

Data are expressed as median (IQR) values

**Fig. 1:**
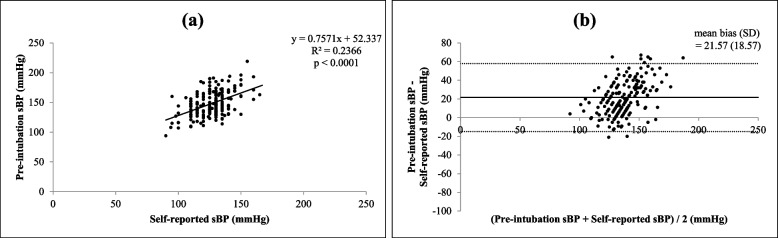
Correlation between self-reported systolic blood pressure (BP) and pre-intubation systolic BP (**a**) (*r* = 0.3970, *p* < 0.0001, 95% confidence interval: CI = 0.2629 to 0.5160) and Bland–Altman plots between self-reported systolic BP and pre-intubation systolic BP (**b**) (mean bias ± standard deviation: SD = 21.5 ± 18.5 mmHg, 95% limits of agreement: LOA = -14.8 to + 57.9 mmHg)

**Fig. 2:**
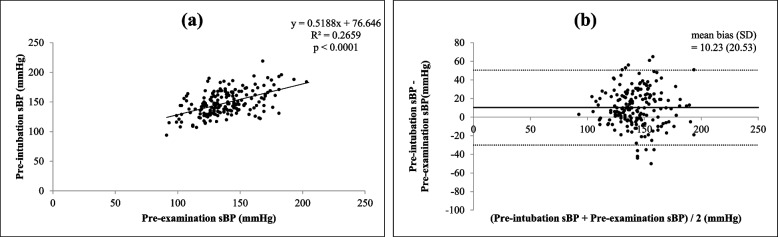
Correlation between pre-examination systolic BP and pre-intubation systolic BP (**a**) (*r* = 0.4673, *p* < 0.0001, 95% CI = 0.3415 to 0.5767) and Bland–Altman plots between pre-examination systolic BP and pre-intubation systolic BP (**b**) (mean bias ± SD = 10.2 ± 20.5 mmHg, 95% LOA = -30.0 to + 50.4 mmHg)

**Fig. 3:**
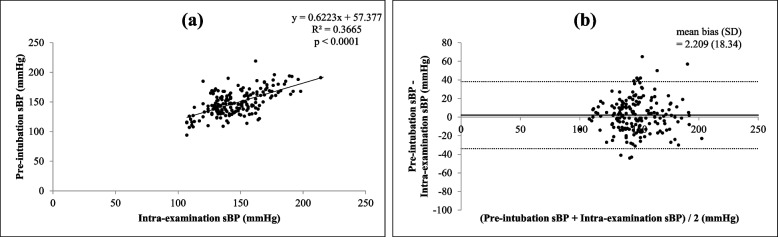
Correlation between intra-examination systolic BP and pre-intubation systolic BP (**a**) (*r* = 0.5230, *p* < 0.0001, 95% CI = 0.4050 to 0.6238) and Bland–Altman plots between intra-examination systolic BP and pre-intubation systolic BP (**b**) (mean bias ± SD = 2.2 ± 18.3 mmHg, 95% LOA = -33.7 to + 38.1 mmHg)

**Fig. 4:**
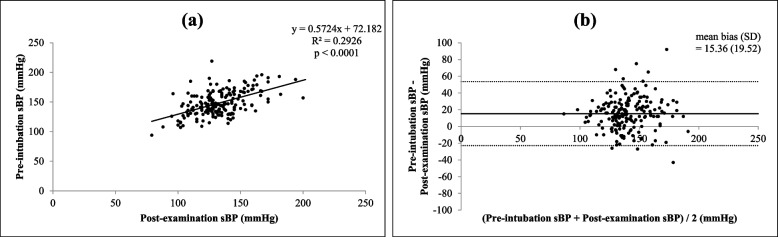
Correlation between post-examination systolic BP and pre-intubation systolic BP (**a**) (*r* = 0.4980, *p* < 0.0001, 95% CI = 0.3764 to 0.6028) and Bland–Altman plots between post-examination systolic BP and pre-intubation systolic BP (**b**) (mean bias ± SD = 15.3 ± 19.5 mmHg, 95% LOA = -22.9 to + 53.6 mmHg)

There were significant correlations (all *p* < 0.0001) between each of the four different times and settings of preoperative systolic BP (self-reported, pre-examination, intra-examination, post-examination) and pre-intubation systolic BP, as shown in Figs. 1(a), 2(a), 3(a) and 4(a)﻿. Intra-operative systolic BP had the strongest correlation with pre-intubation systolic BP (*r* = 0.5230, *p* < 0.0001, 95% CI = 0.4050 to 0.6238). On Bland–Altman analysis of the systolic BPs, the average intra-examination systolic BP was the most reliable for predicting pre-intubation systolic BP, with a smallest mean bias of 2.2 mmHg and a narrowest 95% LOA of + 38.1 to -33.7 mmHg, as shown in Figs. 1(b), 2(b), 3(b) and 4(b).

**Fig. 5:**
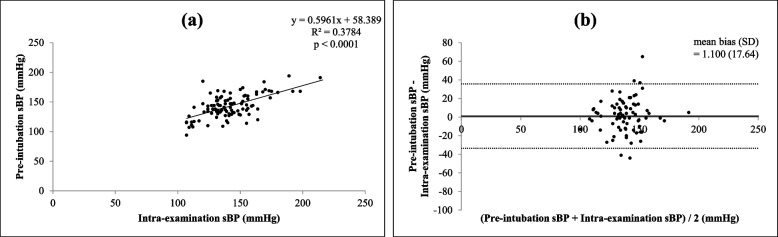
Correlation between intra-examination systolic BP and pre-intubation systolic BP in patients without regular antihypertensive medication (**a**) (*r* = 0.5006, *p* < 0.0001, 95% CI = 0.3321 to 0.6381) and Bland–Altman plots between intra-examination systolic BP and pre-intubation systolic BP in patients without regular antihypertensive medication (**b**) (mean bias ± SD = 1.1 ± 17.6 mmHg, 95% LOA = -33.47 to + 35.67 mmHg)

**Fig. 6:**
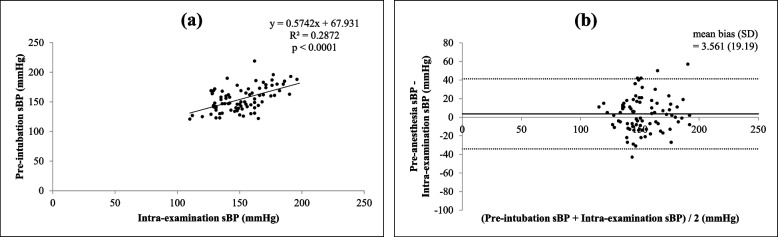
Correlation between intra-examination systolic BP and pre-intubation systolic BP in patients with regular antihypertensive medication (**a**) (*r* = 0.4919, *p* < 0.0001, 95% CI = 0.3018 to 0.6443) and Bland–Altman plots between intra-examination systolic BP and pre-intubation systolic BP in patients with regular antihypertensive medication (**b**) (mean bias ± SD = 3.5 ± 19.1 mmHg, 95% LOA = -34.05 to + 41.17 mmHg)

In patients with or without regular antihypertensive medication before the operation, the relationship between intra-examination systolic BP and pre-intubation systolic BP seemed to be similar and had a significant correlation (both *p* < 0.0001), but it correlated slightly more strongly in patients without regular antihypertensive medication (*r* = 0.5006, 95% CI = 0.3321 to 0.6381) than in patients with regular antihypertensive medication (*r* = 0.4919, 95% CI = 0.3018 to 0.6443), as shown in Figs. 5(a) and 6(a). The results of Bland–Altman analysis were similar as well (without regular medication: mean bias 1.1 mmHg, 95% LOA = -33.47 to + 35.67 mmHg vs with regular medication: mean bias 3.5 mmHg, 95% LOA = -34.05 to + 41.17 mmHg), as shown in Figs. 5(b) and 6(b).

In patients with or without daily BP measurement, there were no significant differences between two measured points or groups (Tables 3 and 4).

**Table 3:** Patients’ self-reported BP measurement settings

Group (Measurement settings)	Number of patients (%)
Group A (At work/hospital admission, irregular)	92 (50.5%)
Group B (At home, regular,)	90 (49.5%)

**Table 4:** Systolic BP in the two groups

	Self-reported BP (on examination sheet)	Pre-examination BP (measured at clinic)
Group A	125/70 (94–130.7/48–80)	134/77 (93–147/49–85.2)
Group B	130/70 (120–135/68–79.5)	138.5/75 (126–153.7/67–81)

Data are expressed as median systolic/diastolic BP (IQR) values (mmHg)

## Discussion

In this study, preoperative systolic BP measured during the anesthesiologist’s examination (intra-examination systolic BP) was found to be closely related to pre-intubation BP measured in the operating room. This result was similar among patients with or without regular antihypertensive medications.

In the clinical setting, it is very important for anesthesiologists to know the precise “baseline BP” for each patient before the induction of anesthesia because this BP is an essential indicator for maintaining adequate systemic hemodynamics in the perioperative period [1–3]. Abnormal preoperative BP is known to affect intra-anesthesia BP, since Bedford et al. [2] reported that hypertension at the time of hospital admission was a predictor of more severe reactive hypertension following rapid-sequence tracheal intubation. Preoperative BP is also related to postoperative morality, since Venkatesan et al. [3] reported that there was a significant association between preoperative diastolic BP values and increased postoperative 30-day mortality in elderly patients. Such multiple reports show the importance of accurately assessing the patients’ BP preoperatively.

Whether pre-intubation BP measured in the operating room adequately represents baseline BP is still controversial. Normal daytime BP is often used as the baseline reference, and there are reports that pre-intubation BP is not similar [5–7]. Saugel et al. [6] evaluated differences between ambulatory and perioperative mean arterial pressures (MAPs) in patients who underwent elective non-cardiac surgery with general anesthesia, and they showed that there was only a weak correlation between the pre-induction and daytime MAPs. They concluded that pre-induction MAP could not be used as a surrogate for the normal daytime MAP. Van Klei et al. [5] reported that the average pre-induction BP was about 10 mmHg higher than the preoperative BP obtained during preoperative evaluation outside the operating room.

In contrast, there have been reports of the importance of assessing pre-intubation BP as the reference. Wax et al. [9] reported that pre-intubation hypertension (BP above 140/90 mmHg) was related to postoperative adverse outcome and found that increasing severity of pre-induction hypertension was an independent risk factor for postoperative myocardial injury/infarction or in-hospital death. In addition, Bijker et al. [1] performed a systematic literature search to identify definitions of intraoperative hypotension that were used in the anesthesiology literature and found that the baseline BP was most frequently based on BP measurements taken immediately before induction of anesthesia. These two reports show that it is still important or useful for many anesthesiologists to assess pre-intubation BP as the baseline BP or as a reference value to evaluate abnormal intraoperative BP. The present study showed that the preoperative BP measured during the examination by anesthesiologists closely resembled the pre-induction BP measured in the operating room. This finding might indicate that the former could be used as a surrogate for pre-induction BP.

BP fluctuation during perioperative anesthetic examinations was observed in the present study. The intra-examination BP measured by anesthesiologists was higher than home-measured BP before examination and pre- or post-examination BP. This phenomenon might be attributable to white-coat hypertension associated with anxiety or stress [4, 8]. The present study confirmed that relatively high BP observed during anesthesiologists’ examinations decreased after the examination, which indicated the elimination of white-coat stress. The present result might indicate that patients during the anesthesiologists’ examination might be in a similar mental condition as on the surgery table before anesthesia. Pre-operative anxiety is a well-known mental condition [10], and there are reports that anxiety increases from the ward to the operating room [11]. It is also common knowledge that anxiety causes hypertension [12]. Indeed, Drummond et al. [8] reported the effect of white-coat hypertension on day-of-surgery BP determinations. We should take note of the times and settings of preoperative BP measurement, since BP fluctuates easily, as shown in the present report. Moreover, BP values during examinations by anesthesiologists may be used to predict pre-intubation hypertension.

The aim of the present study was to identify which time points of BP measurements during anesthesiologist’s examinations, including self-reported values, were the most reliable predictors of pre-intubation BP on the surgery table. The clinical implication of the present study is that assessing only self-reported BP or a single-point check of BP in the preoperative period might lead to the risk of overlooking hidden hypertension or white-coat hypertension. Measuring BP several times, especially during the anesthesiologists’ examinations, could be clinically important to obtain more information on the patient’s condition. Appropriate assessment of BP for each patient at the preoperative examination could help us to plan anesthetic management during the perioperative period.

### Study limitations

Several limitations must be considered in this study. First, it was thought that BP fluctuation would be affected by preoperative medical conditions, such as high age or hypertension. However, no relationship between preoperative medical conditions and BP fluctuation was found (data not shown). This might be attributable to the effects of other factors such as anxiety and other mental or clinical conditions not mentioned in this study.

Second, indicators of mental condition were not evaluated, so the effect of anxiety or other mental condition on BP change during examinations by anesthesiologists could not be considered.

## Conclusions

In conclusion, the preoperative systolic BP values measured during the examination by anesthesiologist was found to be closely related to pre-intubation systolic BP measured in the operating room. Higher BP during the preoperative examination may be a result of anxiety-induced stress or white-coat hypertension. Measuring BP during the anesthesiologist’s examination may be useful for predicting hypertension in the pre-intubation period.

## Data Availability

All data used and analyzed during this study are available from the corresponding author on reasonable request.

## Abbreviations

ASA: American society of anesthesiologists
BP: Blood pressure
CI: Confidence interval
CKD: Chronic kidney disease
DM: Diabetes mellitus
HL: Hyperlipidemia
HR: Heart rate
HT: Hypertension
LOA: Limits of agreement
MAP: Mean arterial pressure
PS: Physical status
sBP: Systolic blood pressure
SD: Standard deviation
STROBE: Strengthening the reporting of observational studies in epidemiology
UMIN-CTR: University hospital medical information network clinical trials registry
